# Hematologic disease and chronic alcohol use independently predict poor prognosis in pyoderma gangrenosum

**DOI:** 10.1016/j.jdin.2025.10.006

**Published:** 2025-10-18

**Authors:** Yang Yang, Zhongrui Xu, Wenyao Chen, Jiao Ning, Xiaolei Su, Guangquan Xu, Mengjiao Li, Jiaqi Wang, Gang Wang, Shuai Shao

**Affiliations:** Department of Dermatology, Xijing Hospital, Fourth Military Medical University, Xi’an, Shaanxi, China

**Keywords:** clinical features, prognosis, pyoderma gangrenosum, risk factors, treatment

*To the Editor:* Pyoderma gangrenosum (PG) is a rare neutrophilic dermatosis characterized by rapidly progressive, painful ulcers and frequent recurrence.^1^^,^^2^ Its erratic course, high relapse rate, and lack of validated biomarkers leave major gaps in management.^3^ Although systemic corticosteroids and immunosuppressants remain first-line,^4^ response is heterogeneous and relapse rates stay high despite aggressive treatment. Identifying clinical features and prognostic risk factors is therefore critical for optimizing patient prognosis.

We analyzed 110 PG patients at Xijing Hospital, China (2008-2024) (Supplementary Fig 1, available via Mendeley at https://data.mendeley.com/datasets/xn5bcs587j/1). Clinical information, treatments, and prognosis were extracted from medical records and assessed using Cox regression. The cohort was predominantly male (65.5%), with a mean onset age of 48 years. Lower extremities were the most common sites of involvement (70%), reflecting both trauma susceptibility and diagnostic challenges in distinguishing PG from vascular ulcers. Mechanical injury or trauma emerged as the most common precipitating factor (37.4%), highlighting the pivotal role of external insults in disease activation. Misdiagnosis occurred in 65.5% of cases, most frequently as vasculitis or infectious ulcers, and led to inappropriate initial management (Supplementary Table I, available via Mendeley at https://data.mendeley.com/datasets/xn5bcs587j/1). Hematologic disease (20%) and inflammatory bowel disease (10.9%) were the 2 most common comorbidities (Supplementary Table II, available via Mendeley at https://data.mendeley.com/datasets/xn5bcs587j/1). Notably, 12.7% of patients had a history of tuberculosis, suggesting a potential but under-recognized link between chronic infection and neutrophilic dysregulation in PG (Table I).

**Table I:** Comparison of clinical characteristics between good prognosis group and poor prognosis group in pyoderma gangrenosum

Clinical characteristics, *n* (%)	Good prognosis group (*n* = 60)	Poor prognosis group (*n* = 50)	*P*
Gender			
Male	39 (65.0)	33 (66.0)	.913
Female	21 (35.0)	17 (34.0)	
Age, mean ± SD	44.28 ± 18.58	46.80 ± 17.94	.474
Disease duration (mo), (IQR)	7.5 (1-36)	12 (3-51)	.099
Chronic alcohol use	6 (10.0)	9 (18.0)	.223
BMI/(kg·m^2^), mean ± SD	23.60 ± 3.88	22.68 ± 4.54	.251
BMI stratified analysis			
BMI <18.5	5 (8.3)	9 (18.0)	.509
18.5 ≤BMI <24	33 (55.0)	24 (48.0)	
24 ≤BMI <28	14 (23.3)	11 (22.0)	
28 ≤BMI	8 (13.3)	6 (12.0)	
Lesion localization			
Lower limbs	42 (70.0)	35 (70.0)	1.000
Upper limb	16 (26.7)	12 (24.0)	.749
Trunk	9 (15.0)	10 (20.0)	.490
Head and neck	7 (11.7)	7 (14.0)	.715
Other (inguinal, perianal, genital)	7 (11.7)	5 (10.0)	.780
Incentive	26 (43.3)	18 (36.0)	.434
Fever	6 (10.0)	9 (18.0)	.223
Past medical history			
Viral hepatitis	5 (8.3)	9 (18.0)	.130
Past history of tuberculosis	7 (11.7)	7 (14.0)	.715
Smoking history	22 (36.7)	14 (28.0)	.335
Surgical history	17 (28.3)	20 (40.0)	.197
Hypertension	12 (20.0)	10 (20.0)	1.000
Hyperlipidemia	4 (6.7)	2 (4.0)	.687
Diabetes mellitus	7 (11.7)	6 (12.0)	.957
Comorbidities			
Inflammatory bowel disease	9 (15.0)	3 (6.0)	.132
Hematological disorders	6 (10.0)	16 (32.0)	.04
Vascular system diseases	8 (13.3)	11 (22.0)	.231
Immune-related diseases	7 (11.7)	8 (16.0)	.510
Laboratory and ancillary tests			
Hypoproteinemia	28 (46.7)	23 (46.0)	.944
Anemia	10 (16.7)	17 (34.0)	.035
Hypokalemia	3 (5.0)	7 (14.0)	.181
Hypocalcemia	3 (5.0)	4 (8.0)	.700
Interstitial lung disease	17 (28.3)	18 (36.0)	.390
Stable time (IQR)	78.5 (22.5-129.75)	6 (3-12)	<.01

*BMI*, Body mass index; *IQR*, interquartile range; *SD*, standard deviation.

All patients received systemic therapy, with corticosteroid monotherapy in 49.1%, corticosteroids plus thalidomide (24.5%) or tumor necrosis factor receptor-Fc fusion protein (10.9%) for refractory cases, and selective addition of cyclosporine or methotrexate. While all achieved remission before discharge, frequent relapses highlight PG's chronic nature and the need for maintenance strategies (Supplementary Table III, available via Mendeley at https://data.mendeley.com/datasets/xn5bcs587j/1).

Multivariate Cox regression identified hematologic disease (hazard ratio 2.42, 95% CI 1.24-4.73) and chronic alcohol use (hazard ratio: 2.70, 95% CI 1.09-6.69) as independent predictors of poor prognosis (Fig 1). Kaplan–Meier survival analyses further demonstrated that patients with underlying hematologic disease or chronic alcohol use had significantly lower cumulative survival probabilities during follow-up (Supplementary Fig 2, available via Mendeley at https://data.mendeley.com/datasets/xn5bcs587j/1). These findings suggest that systemic hematologic disease may exacerbate immune dysregulation and impair wound healing, while alcohol use may further hinder tissue repair via oxidative stress and angiogenesis disruption. Importantly, in patients with active hematologic malignancy, controlling the primary disease must take precedence over escalation of PG-specific therapy.

**Fig 1:**
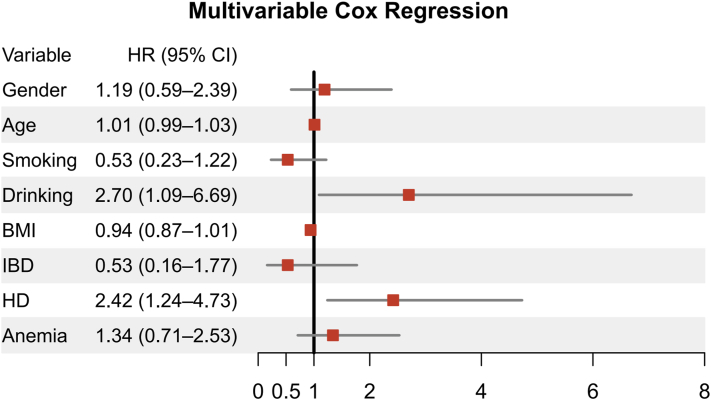
Hazard ratios (HRs) and 95% confidence intervals (CIs) for prognostic factors in pyoderma gangrenosum based on multivariable Cox regression analyses. Multivariate Cox regression adjusted for age, sex, BMI, smoking status, alcohol use, inflammatory bowel disease (IBD), hematologic disease, and anemia. Across multivariate analyses, hematologic disease, and chronic alcohol use emerged as robust predictors of poor prognosis. *BMI*, Body mass index; *HO*, hematologic disease.

This single-center study expands our understanding of PG: the male predominance suggests the influence of genetic or environmental factors; trauma emerges as the leading trigger, necessitating caution during the perioperative period; and the high rate of misdiagnosis by nondermatologists underscores the importance of early dermatology referral and biopsy. Key prognostic findings of hematologic disease and chronic alcohol use support routine blood screening, alcohol counseling, and intensified follow-up. Although the retrospective design and incomplete screening may limit generalizability, the large sample size and long follow-up period provide actionable, real-world insights.

In conclusion, this 16-year study in a Chinese population identifies hematologic disease and chronic alcohol use as risk factors for poor prognosis in PG, supporting early recognition, comorbidity screening, and individualized management to improve prognosis.

## Conflicts of interest

None disclosed.
